# 2-(4-Methyl­phen­yl)-3-oxo-4-phenyl-2,3,3a,4,9,9a-hexa­hydro-1*H*-benzo[*f*]iso­indole-6-carbo­nitrile

**DOI:** 10.1107/S1600536813008568

**Published:** 2013-04-05

**Authors:** Lei Wen, Yimin Hu

**Affiliations:** a School of Chemistry and Materials Science, Anhui Normal University, Wuhu, Anhui 241000, People’s Republic of China

## Abstract

In the title compound, C_26_H_22_N_2_O, one phenyl ring, one five-membered N-heterocyclic ring and one six-membered carbocyclic ring make up the hexa­hydro­benzo[*f*]iso­indole core. Another phenyl group is attached to the heterocyclic N atom as a substituent. The non-aromatic five- and six-membered rings both exhibit boat conformations. In the crystal, weak C—H⋯O and C—H⋯N inter­actions establish the observed three-dimensional structure. The crystal studied was refined as an inversion twin.

## Related literature

For background to domino reactions, see Zhao *et al.* (2012 ▶) and for palladium-catalyzed domino reactions, see Hu *et al.* (2009 ▶, 2010 ▶). For the wide variety of active pharmaceutical ingredients, natural products and other complex organic mol­ecules economically accessible, see: Yu & Hu (2012 ▶); Wang & Hu (2011 ▶). For benzo[*f*]isoindol-1-one derivatives as effective inter­mediates, see: Rixson *et al.* (2012 ▶).
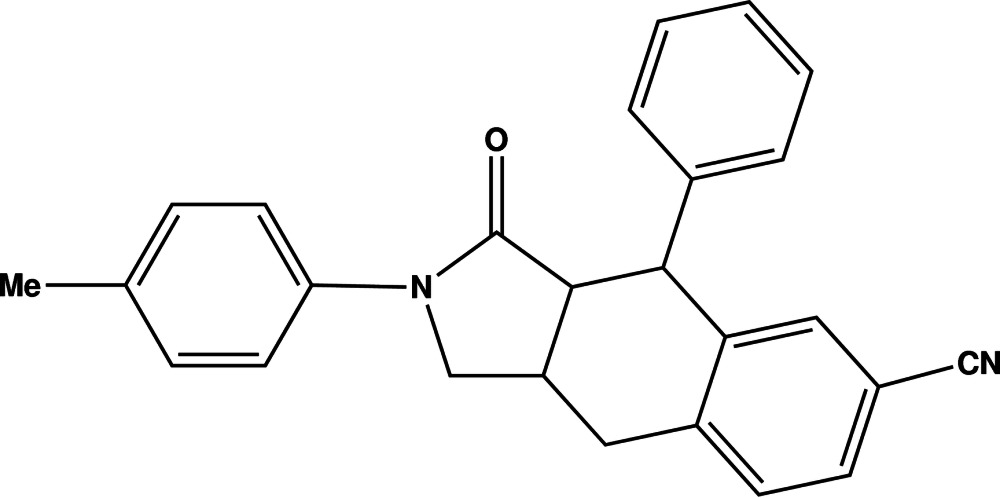


## Experimental

### 

#### Crystal data

C_26_H_22_N_2_O
*M* *_r_* = 378.45
Orthorhombic, 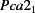

*a* = 25.005 (6) Å
*b* = 5.5023 (14) Å
*c* = 14.790 (4) Å
*V* = 2034.9 (9) Å^3^
*Z* = 4
Mo *K*α radiation
μ = 0.08 mm^−1^
*T* = 291 K
0.28 × 0.24 × 0.22 mm

#### Data collection

Bruker SMART APEX CCD diffractometer
Absorption correction: multi-scan (*SADABS*; Bruker, 2000 ▶) *T*_min_ = 0.972, *T*_max_ = 0.983
14617 measured reflections
4001 independent reflections
2187 reflections with *I* > 2σ(*I*)
*R*_int_ = 0.068

#### Refinement

*R*[*F* ^2^ > 2σ(*F* ^2^)] = 0.052
*wR*(*F* ^2^) = 0.095
*S* = 1.03
4001 reflections
264 parameters
1 restraint
H-atom parameters constrained
Δρ_max_ = 0.13 e Å^−3^
Δρ_min_ = −0.15 e Å^−3^

### 

Data collection: *SMART* (Bruker, 2000 ▶); cell refinement: *SAINT* (Bruker, 2000 ▶); data reduction: *SAINT*; program(s) used to solve structure: *SHELXS97* (Sheldrick, 2008 ▶); program(s) used to refine structure: *SHELXL97* (Sheldrick, 2008 ▶); molecular graphics: *XP* in *SHELXTL* (Sheldrick, 2008 ▶); software used to prepare material for publication: *SHELXL97*.

## Supplementary Material

Crystal structure: contains datablock(s) global, I. DOI: 10.1107/S1600536813008568/im2425sup1.cif

Structure factors: contains datablock(s) I. DOI: 10.1107/S1600536813008568/im2425Isup2.hkl

Supplementary material file. DOI: 10.1107/S1600536813008568/im2425Isup3.cml

Additional supplementary materials:  crystallographic information; 3D view; checkCIF report

## Figures and Tables

**Table 1:** Hydrogen-bond geometry (Å, °)

*D*—H⋯*A*	*D*—H	H⋯*A*	*D*⋯*A*	*D*—H⋯*A*
C25—H25⋯O1^i^	0.93	2.69	3.577 (6)	159
C4—H4⋯N2^ii^	0.93	2.62	3.329 (6)	134
C21—H21⋯O1^iii^	0.93	2.58	3.498 (5)	169

Symmetry codes: (i) 
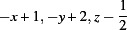
; (ii) 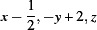; (iii) 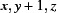.

**Fig. 1.**
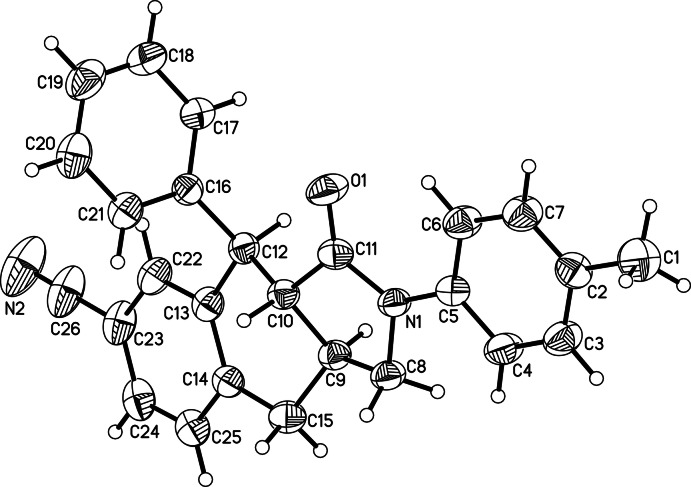
A view of the title compound showing the atom-numbering scheme and displacement ellipsoids drawn at the 30% probability level.

**Fig. 2.**
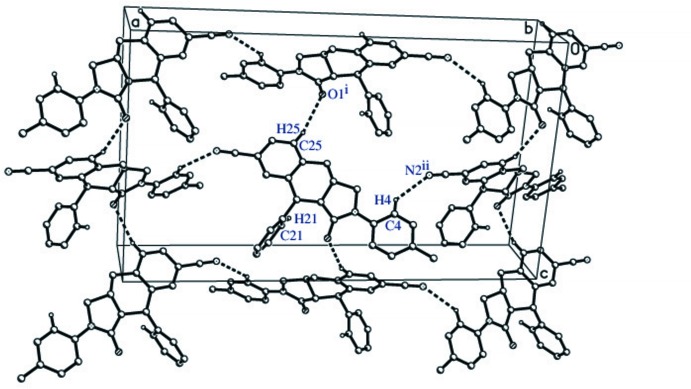
A view of a 2-D layer of the title compound (i: 1 - x,2 - y,-0.5 + z; ii: -0.5 + x,2 - y,z). The interaction C21–H21···O1 producing the 3D-structure was omitted for clarity.

## supplementary crystallographic information

### Comment

Domino reactions have become an important tool of modern organic synthetic chemistry (Zhao *et al.*, 2012). They have made a wide variety of active pharmaceutical ingredients, natural products and other complex organic molecules economically accessible (Yu *et al.*, 2012; Wang *et al.*, 2011). Benzo[*f*]isoindol-1-one derivatives, which have physiological activities themselves, are effective intermediates in the synthesis of many complex natural products (Rixson *et al.*, 2012). We have reported some novel palladium-catalyzed domino reactions of aryl halides with olefins and diynes (Hu *et al.*, 2010; Hu *et al.*, 2009). The reaction of *N*-allyl-3-phenyl-*N*-(*p*-tolyl)acrylamide with 4-bromobenzonitrile, in the presence of palladium(II) acetate and triphenylphosphine in DMF at 413 K for 26 h unexpectedly generated title product.

The crystal structural data of molecule (I), C_26_H_22_N_2_O, reveals that all the bond lengths and angles have normal values. An asymmetric unit is composed of one title compound molecule. The title compound molecule contains one phenyl ring, one five-membered N-heterocyclic ring and one six-membered carbocyclic ring to make up the hexahydro-benzo[*f*]isoindole core. Another phenyl group is attached to nitrogen as a substituent. The non-aromatic five-membered and six-membered ring both show a boat conformation. All the rings are not coplanar (figure 1). In the crystal structure there are weak intermolecular C—H···O and C—H···N interactions (C25—H25 ···O_1_^i ^(i: 1 - *x*,2 - *y*,-0.5 + *z*), C4—H4···N_2_^ii^ (ii: -0.5 + *x*,2 - *y*,*z*) and C21—H21···O_1_^iii^ (iii: *x*,1 + *y*,*z*)) that establish the observed 3D-structure (Figure 2).

### Experimental

An oven-dried Schlenk flask was evacuated, filled with nitrogen, and then charged with *N*-allyl-3-phenyl-*N*-(*p*-tolyl)acrylamide (2.77 g, 10 mmol), 4-bromobenzonitrile (1.82 g, 10 mmol), tributylamine (3 ml), PPh_3_ (52.5 mg, 0.2 mmol), Pd(OAc)_2_ (24 mg, 0.1 mol), and DMF (10 ml) to give a yellow solution. The reaction mixture was heated to 413 K while stirring for 26 hours. The reaction mixture was then cooled to room temperature and the resulting yellow-orange mixture was diluted with Et_2_O (10 ml). The mixture was washed with H_2_O (15 ml) and the aqueous layer was extracted with Et_2_O (20 ml). The combined organic layers were dried (MgSO_4_), filtered, and concentrated *in vacuo*. The crude material was purified by flash column chromatography on silica gel (petroleum ether: EtOAc = 9:1) and recrystallized from EtOAc (yield 3.14 g, 83%). Colorless crystals suitable for X-ray diffraction were obtained by another recrystallization from a solution of the title compound in ethyl acetate over a period of one week.

### Refinement

H atoms were positioned geometrically and refined using a riding model with C—H = 0.93–0.98 Å, and with *U*_iso_(H) = 1.2 (1.5 for methyl groups) times *U*_eq_(C).

Since the crystal obviously was a racemic twin it makes no sense to give information about Flack parameter. So the Flack parameter was omitted from CIF.

### Crystal data

**Table:**

C_26_H_22_N_2_O	*D*_x_ = 1.235 Mg m^−^^3^
*M**_r_* = 378.45	Mo *K*α radiation, λ = 0.71073 Å
Orthorhombic, *P**c**a*2_1_	Cell parameters from 2537 reflections
*a* = 25.005 (6) Å	θ = 2.1–25.4°
*b* = 5.5023 (14) Å	µ = 0.08 mm^−^^1^
*c* = 14.790 (4) Å	*T* = 291 K
*V* = 2034.9 (9) Å^3^	Block, colourless
*Z* = 4	0.28 × 0.24 × 0.22 mm
*F*(000) = 800	

### Data collection

**Table:**

Bruker SMART APEX CCD diffractometer	4001 independent reflections
Radiation source: sealed tube	2187 reflections with *I* > 2σ(*I*)
Graphite monochromator	*R*_int_ = 0.068
phi and ω scans	θ_max_ = 26.0°, θ_min_ = 1.6°
Absorption correction: multi-scan (*SADABS*; Bruker, 2000)	*h* = −30→29
*T*_min_ = 0.972, *T*_max_ = 0.983	*k* = −6→6
14617 measured reflections	*l* = −18→18

### Refinement

**Table:**

Refinement on *F*^2^	1 restraint
Least-squares matrix: full	Hydrogen site location: inferred from neighbouring sites
*R*[*F*^2^ > 2σ(*F*^2^)] = 0.052	H-atom parameters constrained
*wR*(*F*^2^) = 0.095	*w* = 1/[σ^2^(*F*_o_^2^) + (0.030*P*)^2^] where *P* = (*F*_o_^2^ + 2*F*_c_^2^)/3
*S* = 1.03	(Δ/σ)_max_ < 0.001
4001 reflections	Δρ_max_ = 0.13 e Å^−^^3^
264 parameters	Δρ_min_ = −0.15 e Å^−^^3^

### Special details

**Table:**

Geometry. All e.s.d.'s (except the e.s.d. in the dihedral angle between two l.s. planes) are estimated using the full covariance matrix. The cell e.s.d.'s are taken into account individually in the estimation of e.s.d.'s in distances, angles and torsion angles; correlations between e.s.d.'s in cell parameters are only used when they are defined by crystal symmetry. An approximate (isotropic) treatment of cell e.s.d.'s is used for estimating e.s.d.'s involving l.s. planes.
Refinement. Refined as a 2-component inversion twin.

### Fractional atomic coordinates and isotropic or equivalent isotropic displacement parameters (Å^2^)

**Table:**

	*x*	*y*	*z*	*U*_iso_*/*U*_eq_	
C1	0.27416 (18)	−0.1214 (8)	0.8866 (4)	0.0920 (15)	
H1A	0.2484	−0.0456	0.9255	0.138*	
H1B	0.2899	−0.2577	0.9171	0.138*	
H1C	0.2568	−0.1761	0.8323	0.138*	
C2	0.31699 (18)	0.0587 (8)	0.8628 (3)	0.0682 (12)	
C3	0.31038 (18)	0.2255 (8)	0.7939 (3)	0.0762 (13)	
H3	0.2783	0.2264	0.7621	0.091*	
C4	0.34912 (17)	0.3895 (8)	0.7706 (3)	0.0741 (13)	
H4	0.3424	0.5008	0.7247	0.089*	
C5	0.39807 (16)	0.3928 (7)	0.8141 (3)	0.0543 (10)	
C6	0.40556 (18)	0.2282 (8)	0.8835 (3)	0.0690 (13)	
H6	0.4375	0.2277	0.9157	0.083*	
C7	0.36579 (18)	0.0645 (8)	0.9050 (3)	0.0751 (14)	
H7	0.3724	−0.0479	0.9506	0.090*	
C8	0.42905 (15)	0.7232 (7)	0.7095 (3)	0.0594 (11)	
H8A	0.4059	0.8575	0.7261	0.071*	
H8B	0.4135	0.6377	0.6585	0.071*	
C9	0.48494 (15)	0.8106 (7)	0.6881 (2)	0.0525 (10)	
H9	0.5021	0.6863	0.6506	0.063*	
C10	0.51232 (13)	0.8093 (7)	0.7792 (3)	0.0515 (10)	
H10	0.5022	0.9586	0.8110	0.062*	
C11	0.48623 (16)	0.6001 (7)	0.8268 (3)	0.0566 (11)	
C12	0.57264 (14)	0.8097 (7)	0.7688 (2)	0.0515 (10)	
H12	0.5823	0.6549	0.7404	0.062*	
C13	0.58818 (16)	1.0120 (7)	0.7015 (3)	0.0545 (10)	
C14	0.55105 (16)	1.1225 (7)	0.6438 (3)	0.0523 (10)	
C15	0.49270 (16)	1.0529 (7)	0.6417 (3)	0.0640 (11)	
H15A	0.4806	1.0424	0.5795	0.077*	
H15B	0.4717	1.1763	0.6722	0.077*	
C16	0.60352 (16)	0.8242 (7)	0.8567 (3)	0.0520 (10)	
C17	0.63873 (18)	0.6434 (8)	0.8796 (3)	0.0755 (14)	
H17	0.6434	0.5123	0.8407	0.091*	
C18	0.6674 (2)	0.6525 (11)	0.9596 (4)	0.0998 (18)	
H18	0.6907	0.5270	0.9747	0.120*	
C19	0.6615 (2)	0.8440 (12)	1.0156 (4)	0.0884 (16)	
H19	0.6810	0.8514	1.0690	0.106*	
C20	0.62664 (19)	1.0285 (10)	0.9938 (3)	0.0782 (14)	
H20	0.6226	1.1602	1.0326	0.094*	
C21	0.59746 (17)	1.0182 (8)	0.9137 (3)	0.0625 (11)	
H21	0.5739	1.1429	0.8989	0.075*	
C22	0.64117 (16)	1.0829 (8)	0.6969 (3)	0.0624 (11)	
H22	0.6661	1.0069	0.7341	0.075*	
C23	0.65808 (19)	1.2639 (9)	0.6386 (3)	0.0681 (12)	
C24	0.6207 (2)	1.3755 (8)	0.5835 (3)	0.0665 (12)	
H24	0.6314	1.4998	0.5448	0.080*	
C25	0.56853 (19)	1.3048 (7)	0.5855 (3)	0.0625 (12)	
H25	0.5441	1.3796	0.5472	0.075*	
C26	0.7132 (2)	1.3289 (10)	0.6345 (3)	0.0920 (17)	
N1	0.43779 (13)	0.5579 (5)	0.7866 (2)	0.0536 (8)	
N2	0.7571 (2)	1.3807 (9)	0.6321 (3)	0.1284 (19)	
O1	0.50390 (11)	0.4871 (5)	0.8918 (2)	0.0770 (8)	

### Atomic displacement parameters (Å^2^)

**Table:**

	*U*^11^	*U*^22^	*U*^33^	*U*^12^	*U*^13^	*U*^23^
C1	0.078 (3)	0.098 (4)	0.101 (4)	−0.015 (3)	0.014 (3)	0.018 (4)
C2	0.070 (3)	0.066 (3)	0.069 (3)	−0.002 (2)	0.008 (3)	0.006 (3)
C3	0.058 (3)	0.088 (3)	0.083 (3)	−0.009 (3)	−0.010 (3)	0.022 (3)
C4	0.056 (3)	0.079 (3)	0.087 (3)	−0.005 (3)	−0.012 (3)	0.027 (3)
C5	0.055 (3)	0.055 (3)	0.053 (2)	0.005 (2)	0.000 (2)	0.004 (2)
C6	0.066 (3)	0.079 (3)	0.062 (3)	−0.001 (3)	−0.004 (2)	0.016 (3)
C7	0.079 (3)	0.083 (4)	0.063 (3)	−0.007 (3)	−0.003 (3)	0.022 (3)
C8	0.057 (3)	0.063 (3)	0.058 (3)	−0.001 (2)	−0.009 (2)	0.014 (2)
C9	0.055 (3)	0.056 (2)	0.046 (2)	0.003 (2)	−0.003 (2)	0.003 (2)
C10	0.050 (2)	0.052 (2)	0.052 (3)	0.005 (2)	−0.002 (2)	0.000 (2)
C11	0.054 (3)	0.060 (3)	0.055 (3)	0.009 (2)	0.002 (2)	0.003 (2)
C12	0.054 (2)	0.052 (2)	0.048 (2)	0.003 (2)	−0.002 (2)	−0.006 (2)
C13	0.060 (3)	0.061 (3)	0.042 (2)	0.002 (2)	0.004 (2)	−0.010 (2)
C14	0.060 (3)	0.050 (2)	0.046 (2)	0.002 (2)	0.002 (2)	−0.001 (2)
C15	0.070 (3)	0.067 (3)	0.055 (2)	0.004 (2)	−0.001 (2)	0.010 (2)
C16	0.053 (3)	0.051 (3)	0.052 (3)	−0.003 (2)	0.000 (2)	0.003 (2)
C17	0.081 (3)	0.062 (3)	0.083 (4)	0.010 (2)	−0.028 (3)	−0.003 (3)
C18	0.101 (4)	0.088 (4)	0.111 (5)	−0.005 (3)	−0.050 (4)	0.022 (4)
C19	0.091 (4)	0.112 (5)	0.063 (3)	−0.022 (4)	−0.018 (3)	0.018 (4)
C20	0.086 (4)	0.098 (4)	0.051 (3)	−0.017 (3)	0.009 (3)	−0.016 (3)
C21	0.067 (3)	0.074 (3)	0.047 (3)	0.000 (2)	0.004 (2)	−0.007 (2)
C22	0.059 (3)	0.082 (3)	0.046 (2)	−0.005 (2)	0.001 (2)	−0.007 (2)
C23	0.073 (3)	0.092 (3)	0.039 (2)	−0.023 (3)	0.011 (2)	−0.011 (3)
C24	0.092 (4)	0.066 (3)	0.042 (2)	−0.018 (3)	0.014 (3)	−0.008 (2)
C25	0.079 (3)	0.062 (3)	0.046 (3)	−0.004 (3)	0.008 (2)	−0.001 (2)
C26	0.094 (4)	0.140 (5)	0.042 (3)	−0.050 (4)	0.004 (3)	−0.001 (3)
N1	0.051 (2)	0.055 (2)	0.055 (2)	0.0007 (17)	−0.0069 (18)	0.0107 (17)
N2	0.110 (4)	0.214 (5)	0.061 (3)	−0.072 (4)	−0.001 (3)	0.005 (4)
O1	0.0645 (17)	0.096 (2)	0.0705 (19)	−0.0025 (16)	−0.0153 (17)	0.0313 (19)

### Geometric parameters (Å, º)

**Table:**

C1—C2	1.501 (5)	C12—C13	1.543 (5)
C1—H1A	0.9600	C12—H12	0.9800
C1—H1B	0.9600	C13—C22	1.383 (5)
C1—H1C	0.9600	C13—C14	1.400 (5)
C2—C7	1.372 (5)	C14—C25	1.393 (5)
C2—C3	1.380 (6)	C14—C15	1.509 (5)
C3—C4	1.368 (6)	C15—H15A	0.9700
C3—H3	0.9300	C15—H15B	0.9700
C4—C5	1.383 (5)	C16—C21	1.369 (5)
C4—H4	0.9300	C16—C17	1.371 (5)
C5—C6	1.381 (5)	C17—C18	1.385 (6)
C5—N1	1.406 (5)	C17—H17	0.9300
C6—C7	1.379 (5)	C18—C19	1.348 (7)
C6—H6	0.9300	C18—H18	0.9300
C7—H7	0.9300	C19—C20	1.376 (6)
C8—N1	1.475 (5)	C19—H19	0.9300
C8—C9	1.512 (5)	C20—C21	1.393 (6)
C8—H8A	0.9700	C20—H20	0.9300
C8—H8B	0.9700	C21—H21	0.9300
C9—C15	1.512 (5)	C22—C23	1.384 (6)
C9—C10	1.512 (5)	C22—H22	0.9300
C9—H9	0.9800	C23—C24	1.383 (6)
C10—C11	1.499 (5)	C23—C26	1.424 (6)
C10—C12	1.516 (5)	C24—C25	1.363 (5)
C10—H10	0.9800	C24—H24	0.9300
C11—O1	1.227 (5)	C25—H25	0.9300
C11—N1	1.369 (5)	C26—N2	1.136 (6)
C12—C16	1.514 (5)		
			
C2—C1—H1A	109.5	C10—C12—H12	106.8
C2—C1—H1B	109.5	C13—C12—H12	106.8
H1A—C1—H1B	109.5	C22—C13—C14	118.9 (4)
C2—C1—H1C	109.5	C22—C13—C12	118.4 (4)
H1A—C1—H1C	109.5	C14—C13—C12	122.6 (3)
H1B—C1—H1C	109.5	C25—C14—C13	118.8 (4)
C7—C2—C3	115.3 (4)	C25—C14—C15	118.3 (4)
C7—C2—C1	122.9 (4)	C13—C14—C15	122.9 (3)
C3—C2—C1	121.8 (4)	C14—C15—C9	109.8 (3)
C4—C3—C2	122.7 (4)	C14—C15—H15A	109.7
C4—C3—H3	118.7	C9—C15—H15A	109.7
C2—C3—H3	118.7	C14—C15—H15B	109.7
C3—C4—C5	121.2 (4)	C9—C15—H15B	109.7
C3—C4—H4	119.4	H15A—C15—H15B	108.2
C5—C4—H4	119.4	C21—C16—C17	119.0 (4)
C6—C5—C4	117.2 (4)	C21—C16—C12	120.9 (4)
C6—C5—N1	122.9 (4)	C17—C16—C12	120.1 (4)
C4—C5—N1	119.9 (4)	C16—C17—C18	121.2 (5)
C7—C6—C5	120.1 (4)	C16—C17—H17	119.4
C7—C6—H6	119.9	C18—C17—H17	119.4
C5—C6—H6	119.9	C19—C18—C17	119.7 (5)
C2—C7—C6	123.4 (4)	C19—C18—H18	120.2
C2—C7—H7	118.3	C17—C18—H18	120.2
C6—C7—H7	118.3	C18—C19—C20	120.2 (5)
N1—C8—C9	102.8 (3)	C18—C19—H19	119.9
N1—C8—H8A	111.2	C20—C19—H19	119.9
C9—C8—H8A	111.2	C19—C20—C21	120.0 (5)
N1—C8—H8B	111.2	C19—C20—H20	120.0
C9—C8—H8B	111.2	C21—C20—H20	120.0
H8A—C8—H8B	109.1	C16—C21—C20	119.9 (4)
C8—C9—C15	119.6 (3)	C16—C21—H21	120.1
C8—C9—C10	103.3 (3)	C20—C21—H21	120.1
C15—C9—C10	110.5 (3)	C13—C22—C23	121.7 (4)
C8—C9—H9	107.6	C13—C22—H22	119.1
C15—C9—H9	107.6	C23—C22—H22	119.1
C10—C9—H9	107.6	C24—C23—C22	118.7 (4)
C11—C10—C9	103.0 (3)	C24—C23—C26	121.1 (5)
C11—C10—C12	118.8 (3)	C22—C23—C26	120.2 (5)
C9—C10—C12	111.1 (3)	C25—C24—C23	120.4 (4)
C11—C10—H10	107.8	C25—C24—H24	119.8
C9—C10—H10	107.8	C23—C24—H24	119.8
C12—C10—H10	107.8	C24—C25—C14	121.3 (4)
O1—C11—N1	125.0 (4)	C24—C25—H25	119.3
O1—C11—C10	126.9 (4)	C14—C25—H25	119.3
N1—C11—C10	108.1 (3)	N2—C26—C23	179.3 (5)
C16—C12—C10	114.8 (3)	C11—N1—C5	127.5 (3)
C16—C12—C13	112.8 (3)	C11—N1—C8	111.3 (3)
C10—C12—C13	108.5 (3)	C5—N1—C8	121.2 (3)
C16—C12—H12	106.8		
			
C7—C2—C3—C4	−1.5 (7)	C8—C9—C15—C14	169.1 (4)
C1—C2—C3—C4	−179.3 (5)	C10—C9—C15—C14	49.4 (4)
C2—C3—C4—C5	1.5 (7)	C10—C12—C16—C21	−60.2 (5)
C3—C4—C5—C6	−1.7 (6)	C13—C12—C16—C21	64.8 (4)
C3—C4—C5—N1	177.8 (4)	C10—C12—C16—C17	120.8 (4)
C4—C5—C6—C7	2.0 (6)	C13—C12—C16—C17	−114.2 (4)
N1—C5—C6—C7	−177.5 (4)	C21—C16—C17—C18	1.0 (7)
C3—C2—C7—C6	1.9 (7)	C12—C16—C17—C18	−180.0 (4)
C1—C2—C7—C6	179.6 (4)	C16—C17—C18—C19	−1.1 (8)
C5—C6—C7—C2	−2.2 (7)	C17—C18—C19—C20	0.7 (8)
N1—C8—C9—C15	−154.6 (3)	C18—C19—C20—C21	−0.1 (7)
N1—C8—C9—C10	−31.4 (4)	C17—C16—C21—C20	−0.4 (6)
C8—C9—C10—C11	32.8 (4)	C12—C16—C21—C20	−179.5 (4)
C15—C9—C10—C11	161.9 (3)	C19—C20—C21—C16	0.0 (6)
C8—C9—C10—C12	161.0 (3)	C14—C13—C22—C23	1.7 (6)
C15—C9—C10—C12	−69.8 (4)	C12—C13—C22—C23	−179.7 (4)
C9—C10—C11—O1	159.3 (4)	C13—C22—C23—C24	−0.1 (6)
C12—C10—C11—O1	36.0 (6)	C13—C22—C23—C26	−178.8 (4)
C9—C10—C11—N1	−22.2 (4)	C22—C23—C24—C25	−1.3 (6)
C12—C10—C11—N1	−145.5 (3)	C26—C23—C24—C25	177.3 (4)
C11—C10—C12—C16	−64.4 (5)	C23—C24—C25—C14	1.2 (6)
C9—C10—C12—C16	176.4 (3)	C13—C14—C25—C24	0.3 (6)
C11—C10—C12—C13	168.4 (3)	C15—C14—C25—C24	−179.6 (4)
C9—C10—C12—C13	49.2 (4)	O1—C11—N1—C5	4.4 (6)
C16—C12—C13—C22	37.2 (5)	C10—C11—N1—C5	−174.2 (3)
C10—C12—C13—C22	165.6 (3)	O1—C11—N1—C8	−179.2 (4)
C16—C12—C13—C14	−144.2 (4)	C10—C11—N1—C8	2.2 (4)
C10—C12—C13—C14	−15.8 (5)	C6—C5—N1—C11	−6.8 (6)
C22—C13—C14—C25	−1.7 (6)	C4—C5—N1—C11	173.8 (4)
C12—C13—C14—C25	179.7 (3)	C6—C5—N1—C8	177.2 (4)
C22—C13—C14—C15	178.2 (4)	C4—C5—N1—C8	−2.3 (5)
C12—C13—C14—C15	−0.4 (6)	C9—C8—N1—C11	18.7 (4)
C25—C14—C15—C9	163.7 (3)	C9—C8—N1—C5	−164.7 (3)
C13—C14—C15—C9	−16.2 (5)		

### Hydrogen-bond geometry (Å, º)

**Table:**

*D*—H···*A*	*D*—H	H···*A*	*D*···*A*	*D*—H···*A*
C25—H25···O1^i^	0.93	2.69	3.577 (6)	159
C4—H4···N2^ii^	0.93	2.62	3.329 (6)	134
C21—H21···O1^iii^	0.93	2.58	3.498 (5)	169

Symmetry codes: (i) −*x*+1, −*y*+2, *z*−1/2; (ii) *x*−1/2, −*y*+2, *z*; (iii) *x*, *y*+1, *z*.
